# School refusal: mapping the literature by bibliometric analysis

**DOI:** 10.3389/fpsyg.2024.1265781

**Published:** 2024-02-12

**Authors:** Sümeyye Ulaş, Carolina Gonzálvez, İsmail Seçer

**Affiliations:** ^1^Department of Psychological Counseling and Guidance, Atatürk University, Erzurum, Türkiye; ^2^Department of Developmental Psychology and Didactics, Alicante University, Alicante, Spain

**Keywords:** school attendance problem, school refusal, bibliometric analysis, research trends, scientific collaboration

## Abstract

School refusal is considered a risk factor for academic, social, and personal situations, such as school dropouts. Studies have been carried out on school refusal for almost 50 years. However, general research trends have not been mapped yet. This study summarizes the bibliometric analysis of scientific collaborations and prevalence across locations by country and institution, leading researchers, journals, and trends (keywords) in school refusal research. The United States, Japan, Spain, and England are the countries that stand out in terms of school refusal. It can be said that the Journal of American Academy of Child and Adolescent Psychiatry, Cognitive and Behavioral Practice, and Frontiers in Psychology are important journals that publish on school refusal. Researchers named Christopher A. Kearney, Carolina Gonzálvez, Jose Manuel Garcia-Fernandez, David A. Heyne, and Brigit M. Van Widenfelt have been found to have more intensive studies and collaborations on school refusal. The authors keywords common use for school refusal; are truancy, school absenteeism, adolescence, school attendance, school phobia, autism spectrum disorder, and bullying. The findings show that school refusal is a current research area, and scientific collaborations continue to be established. The findings reveal all the details of the school refusal research.

## 1 Introduction

The right to education is widely recognized in various legislative areas at the international, national, regional, and local levels. However, the rate of students who, by their own decision or by external factors, do not attend school regularly is worrying. Because of this, it is a challenge for educational systems and political actions to prevent and eradicate school absenteeism and school dropout.

School refusal behaviors have always occurred, but there has been an increased amount of research on this issue over the last few decades of the twentieth century (Inglés et al., 2015; García-Fernández et al., 2016; Elliot and Place, 2019). Such a long historical focus is partially because School Attendance Problems (SAPs) are related to long- and short-term negative consequences. School absenteeism affects students’ performance in the short term but the social, economic, and health domains in the long term (Ansari et al., 2020).

According to a recent theoretical review, Kearney and Gonzálvez (2022) reformulate the way of understanding and addressing school attendance and related issues claiming the need to forget old-fashioned historical methods and make use of more inclusive postmodern approaches. Traditionally, SAPs were defined as those students physically absent in a specific school building on a specific day (Gentle-Genitty et al., 2020). However, according to the postmodern approach, SAPs should be redefined considering wider and more flexible referrals of this issue, as well as a continuum of SAPs regarding the level of severity (Kearney and Gonzálvez, 2022).

Along the same lines, research on SAPs requires going beyond research from individual concerns and concentration on particular contexts (Kearney, 2021), to foster the creation of collaborations and shared alliances between professionals of different fields and different cultures. Nowadays, studies examining the SAPs are carried out in many fields (child development, social work, criminal and juvenile justice, education, psychiatry, psychology, sociology, school counseling, among others) creating a rich and comprehensive knowledge over the past century on SAPs (Elliot and Place, 2019).

### 1.1 Globalization of science on school attendance problems

Science is increasingly global. The advancement of international projects, global co-authorship networks, international academic conferences, global talent mobility networks and collaboration networks have promoted the globalization of science (Gui et al., 2019). To illustrate this point, in the field of SAPs the International Network for School Attendance (INSA) have served to promote the study of SAPs worldwide, fostering, in turn, collaboration networks between countries around the world.

In view of the large number of articles published annually on SAPs, theoretical reviews, bibliometric studies, and meta-analysis are useful to report greater knowledge about the impact and visibility of scientific production (Gubbels et al., 2019; Eklund et al., 2022). Gradually more studies are carried out internationally on SAPs. Nevertheless, the number of studies on the globalization of science from the aspect of international scientific collaboration is limited.

### 1.2 The present study

The last five decades of research into school attendance and its problems have produced an extensive body of knowledge, as evidenced by simple bibliometric analysis and theoretical reviews (Elliot and Place, 2019). Despite the body of knowledge that has been built up, it is difficult to find exercises of multifaceted bibliometric studies that establish co-citation and bibliographic coupling networks of studies, journals, and researchers. Considering that previous studies warn of an increase in the number of studies over time, as well as a greater representation of countries where the study of school attendance and related issues is a recurring theme, it is time to analyze bibliometric networks on SAPs. Moving from simple scientific production indicators to visualizing bibliometric networks is requested.

Making use of the bibliographical data obtained from the WoS database, the present study employs network visualization analysis to examine the structure, dynamics, and determinants of international scientific collaboration networks on school refusal for the years between 1970 and the present. This bibliometric review will focus on school refusal, one condition under the umbrella term of SAPs, which refers to any problematic absence from school for explained or unexplained reasons (Heyne et al., 2019). The present study aims to determine scientific collaborations on school refusal, analyze the frequency of school refusal studies across locations by countries and organizations, identify leading researchers, and report trends in school refusal studies by making use of the bibliometric analysis with VOSviewer program.

## 2 Materials and methods

In this study, a bibliometric analysis was carried out aiming to determine the current trends of studies on school refusal and scientific collaborations on this subject.

### 2.1 Data sources and data collection

In the present study, data sources were reviewed first. In this context, a search was conducted with the keyword of “school refusal” in the Web of Science (WoS) database. The reason for using the WoS database in this study is that, as a result of the comparison of WoS and other databases, the Wos database has many search options, full recode access to the publications (title, author, sources, abstract, author keywords, cited references, etc.), the existence of refined and analysis options, h-index, and the presence of journals with high impact factors, etc., are evaluated to be more prestigious (Joshi, 2016). The review process was completed on 24.12.2023. All years, languages, document types and indexes were included during the search. The keyword was searched in the topic. In this context, 622 publications were reached (search link: https://www.webofscience.com/wos/woscc/summary/5e74cdc9-ee5a-41c2-bbe5-db0c979368fd-bfb2b6ca/relevance/1).

### 2.2 Data analysis

Data examined in this study was obtained from WoS and then analyzed bibliometrically. Although there is a wide variety of software for bibliometric analysis, VosViewer software is recommended because it allows the highest number of and type analyses in the literature (Van Eck et al., 2008). Therefore, within the scope of this study, a co-occurrence network for keywords, and a bibliographic coupling network for authors, organizations, journals (sources), and countries were visualized using Vosviewer.

## 3 Results

### 3.1 Annual distribution of research

**Figure 1:**
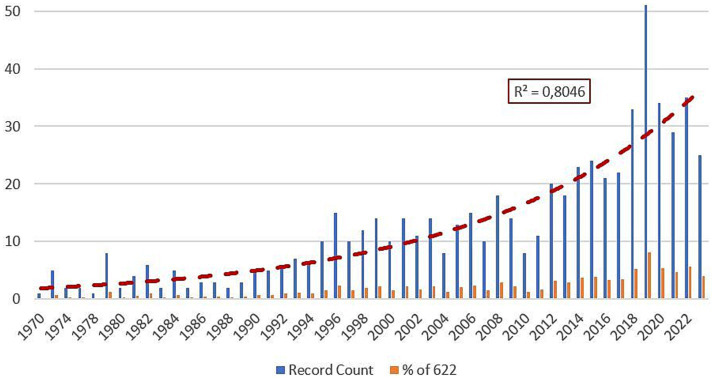
The annual distribution of research.

The findings obtained as a result of the search made on the WoS in order to identify the annual distribution of studies in the field of school refusal (SR) are presented in Figure 1.

According to the data obtained from the WoS, the first study on school refusal was in 1970. Until 1995, the number of publications varied between 1 and 5 (except in 1979). While the publication rate until 1995 was 12%, it reached 31% between 1996 and 2011. In 2012 and 2023, the publication rate was found to be 57%. As a result, school refusal has been handled more intensively by researchers, especially, in the last 12 years.

### 3.2 Scientific cooperation between countries

**Figure 2:**
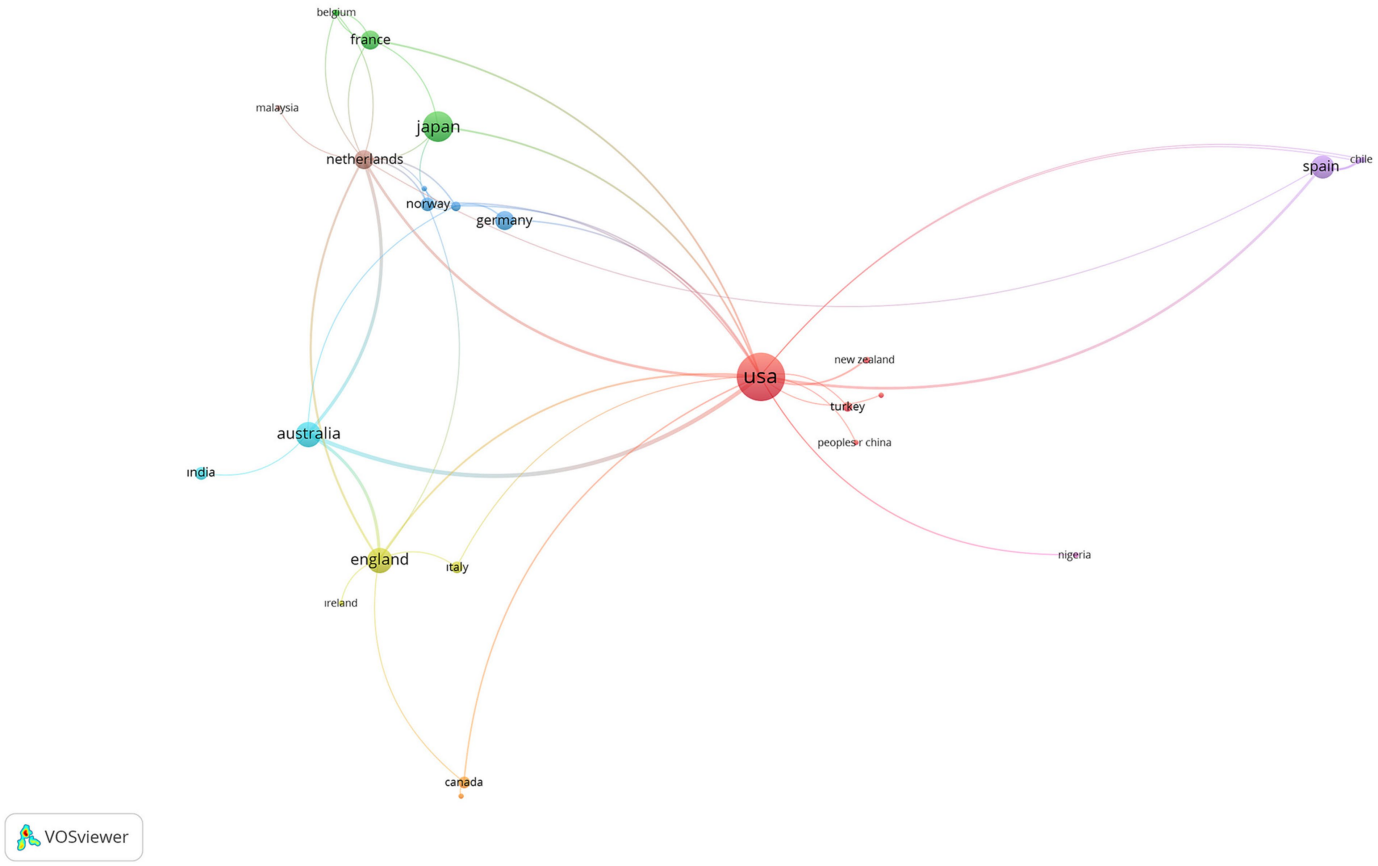
The visualization of cross-country networks on SR.

**Figure 3:**
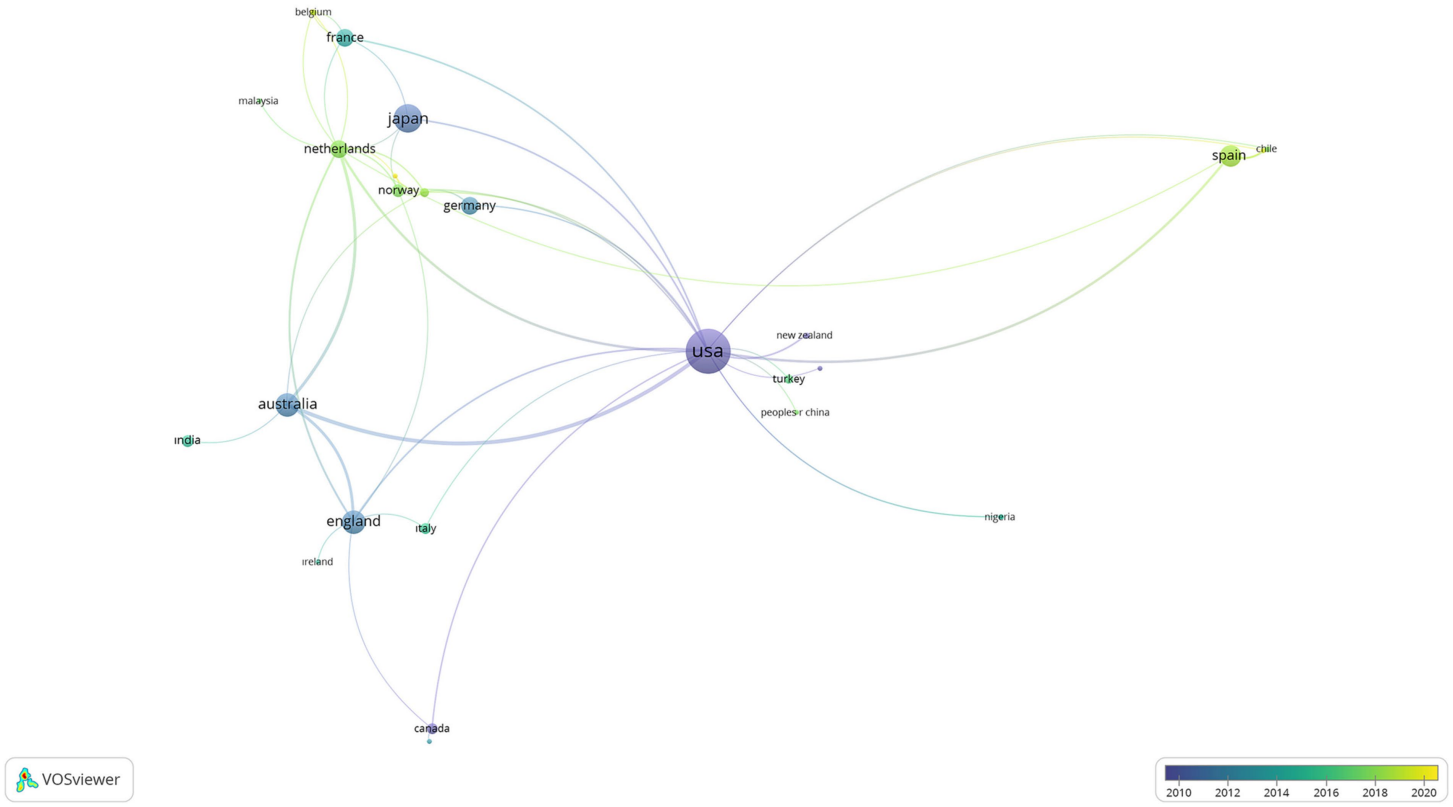
The visualization of cross-country overlay on SR.

In the analysis conducted to determine scientific cooperation between countries on SR,

- The type of analysis: co-authorship,
- A unit of analysis: countries,
- Counting method: full counting method,
- The minimum number of documents of a country: 1,
- The minimum number of citations of a country: 1.

were determined. In the mapping process, the document was used as the weighting criterion of the images. With the limitations made, 27 scientific collaborations from 49 countries were visualized and presented in Figures 2, 3.

According to Figures 2, 3, New Zealand (l:1, tls:3, doc:4), China (l:1, tls:1, doc:3), Turkey (l:1, tls:1, d:8), United States (l:19, tls:55, d:188), Venezuela (l:1, tls:1, doc:1) countries are in the first cluster; Belgium (l:3, tls:3, doc:4), Denmark (l:3, tls:3, doc:3), France (l:5, tls:7, doc:30), Japan (l:4, tls:6, doc:76) countries are in the second cluster; Finland (l:3, tls:4, doc:2), Germany (l:2, tls:4, doc:31), Norway (l:6, tls:8, doc:17), Sweden (l: 6, tls:10, doc:8) are in the third cluster; England (l:7, tls:19, doc:53), Ireland (l:1, tls:1, doc:3), Italy (l:2, tls:2, doc:11) are in the fourth cluster; Chile (l:3, tls:6, doc:4), Ecuador (l:3, tls:5, doc:3), Spain (l:4, tls:14, doc:46) are in the fifth cluster; Australia (l:5, tls:34, doc:53), India (l:1, tls:1, doc:14) are in the sixth cluster; Canada (l:3, tls:4, doc:11) and Philippines (l:1, tls:1, doc:1) are in the seventh cluster; Malaysia (l:1, tls:1, doc:3), Netherlands (l:12, tls:30, doc:30) are in the eighth cluster; Nigeria (l:2, tls:2, doc:2) and South Africa (l:2, tls:2, doc:2) are in the nineth cluster.

As seen in Figure 2, it can be stated that the United States, Japan, Spain, Australia, and England are the countries that stand out from the aspect of school refusal. Examining Figure 3, it can be said that Norway, Netherlands, Belgium, Turkey, Chile, Italy, India, China, and Spain tend to form up-to-date scientific collaborations.

### 3.3 Scientific cooperation between organizations

**Figure 4:**
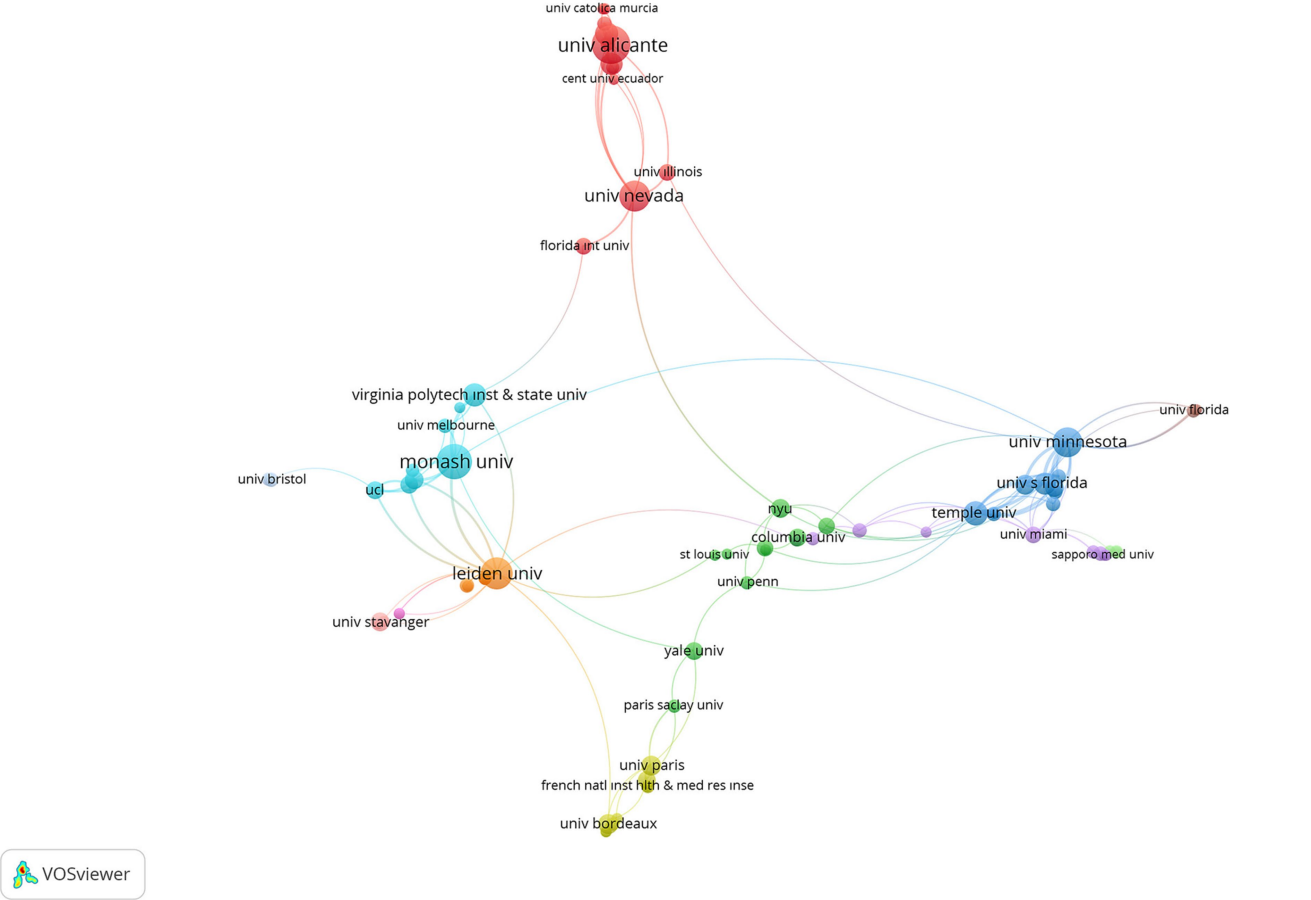
The visualization of cross-organizational networks on SR.

**Figure 5:**
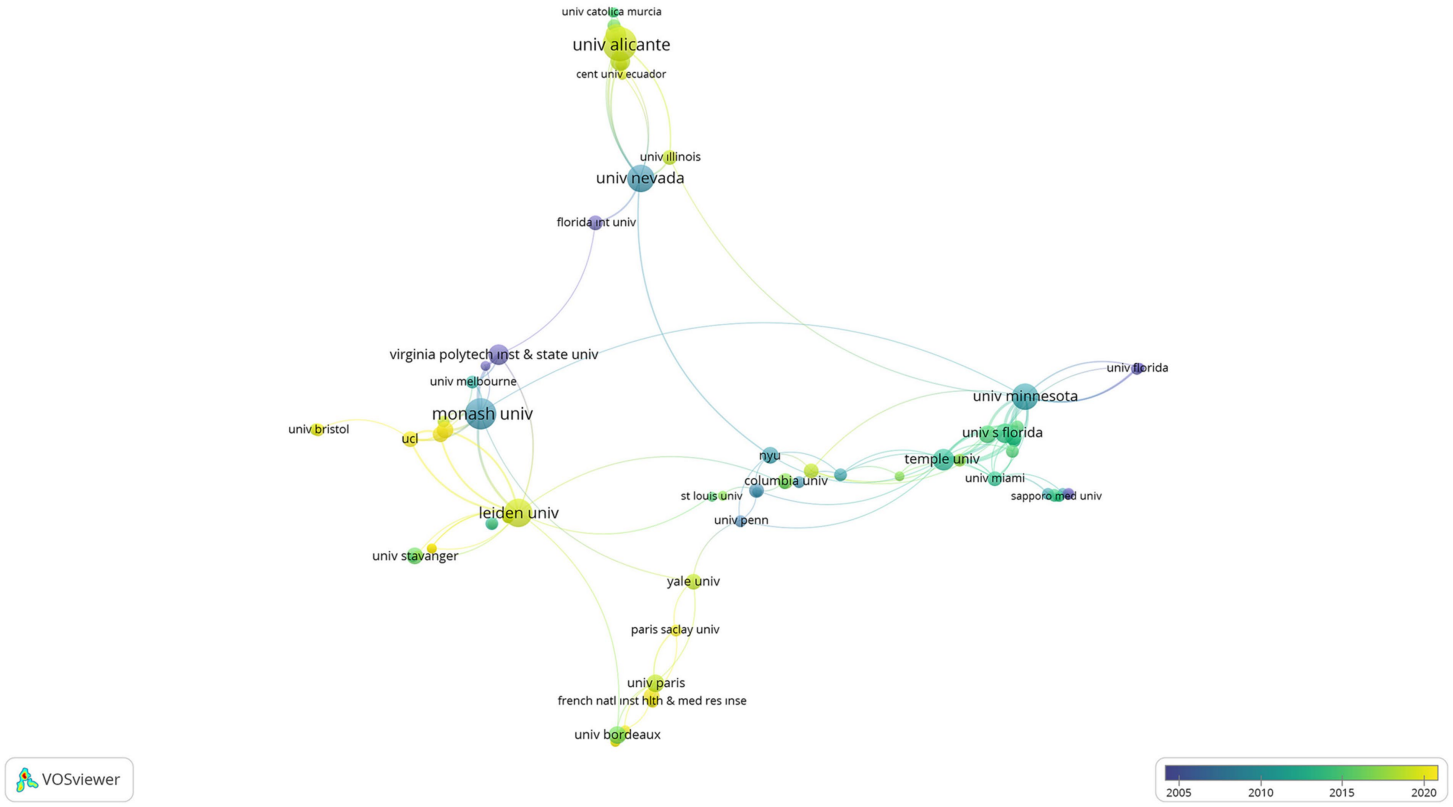
The visualization of cross-organizational overlay on SR.

In the analysis examining the relationship patterns of studies on school refusal according to organizations,

- Type of analysis: co-authorship,
- The unit of analysis: organizations,
- Counting method: full counting method,
- The min. Number of document of an organization: 2,
- The min. Number of citations of an organization: 10.

was determined as. In the mapping process, citations were used as the weighting criterion of the images. With the limitations made, 80 scientific collaborations from 650 organizations were visualized and presented in Figures 4, 5.

According to Figure 4, in all these organizations, University of Alicante, University Nevada, Virginia Polytech Institute and State University, Monash University, University Minnesota, Florida International University, Leiden University, University of Pennsylvania, Miguel Hernandez University of Elche Spain, Temple University, New York University, University Utrecht, University Stavanger, John Hopkins University organizations were found to be at the forefront of SR. Also, according to Figure 5, University of Alicante, Central University of Ecuador, University Illinois, University Bristol, Leiden University, University College London, Monash University, French Institute of Health and Medical Research Institute, Paris Saclay University, Yale University, and University Paris has come to the fore in recent years at the point of establishing scientific collaborations. In addition, citations to research related to SR are presented in Table 1 by organizations.

**Table 1:** Citation numbers of organization.

Cluster	Organization	Number of citation
1. Cluster	University Nevada	1,358
	University of Alicante	526
	Florida International University	472
	Miguel Hernandez University of Elche Spain	352
	University Catolica Murcia	123
	University Illinois	97
	Central University of Ecuador	58
	University of the Bio Bio	27
2. Cluster	University of Pennsylvania	319
	New York University	298
	John Hopkins University	165
	University of Saint Louis.	152
	University of Texas at Austin	117
	Icahn School of Medicine at Mount Sinai	80
	Yale University	51
	Kennedy Krieger Institute	45
	Columbia University	24
	Paris Saclay University	11
3. Cluster	University Minnetsota	648
	Temple University	303
	Vanderbilt University	138
	University of South Florida	108
	Rutgers State University	91
	Griffith University	59
	13th Judical Circuit	57
	University of Souther Mississippi	52
	Wayne State Univ	50
4. Cluster	University Bordeaux	119
	University Paris	93
	Cochin Hospital	41
	Vrije University Brussel	40
	French Institute of Health and Medical Research	32
	Aarhus University	23
	Argenteuil Hospital Center	20
	Saint-Denis Hospital	19
	Salvator Hospital	16
	Toulouse University Hospital (CHU-Toulouse)	13
5. Cluster	Boston University	81
	University Pittsburgh	80
	Arizona State University	80
	University Miami	74
	Penn State University	54
	Sappora Medicine University	54
	Kyushu University	53	Cluster	Organization	Number of citation	
	University Connecticut	35
	Shiga University Medicine Science	16
6. Cluster	Monash University	824
	Virginia Polytech Institute and State University	772
	University Melbourne	117
	Monash Health	38
	University Warwick	37
	Deakin University	33
	University College London	27
	Monash Medical Center	11
7. Cluster	Leiden University	506
	University Utrecht	249
	University Amsterdam	123
	Radboud University Nijmegen	29
	University New England	20
	Kyushu University Health and Welfare	13
8. Cluster	Nova Southeastern University	164
	University Florida	139
	Neuropsychiatry Research Institute	121
	Veteran’s Administration Medical Center	118
9. Cluster	Magelungen Utveckling AB	13
	Lund University	13
	Abo Akad University	13
10. Cluster	University Stavanger	287
	Norweigan University Science and Technology	63
	University Durham	56
11. Cluster	University Hawaii Manoa	56
	University Mississippi	13
	University of Tulsa	42
12. Cluster	University Bristol	142
	University Exeter	141

### 3.4 Journals publishing SR studies

**Figure 6:**
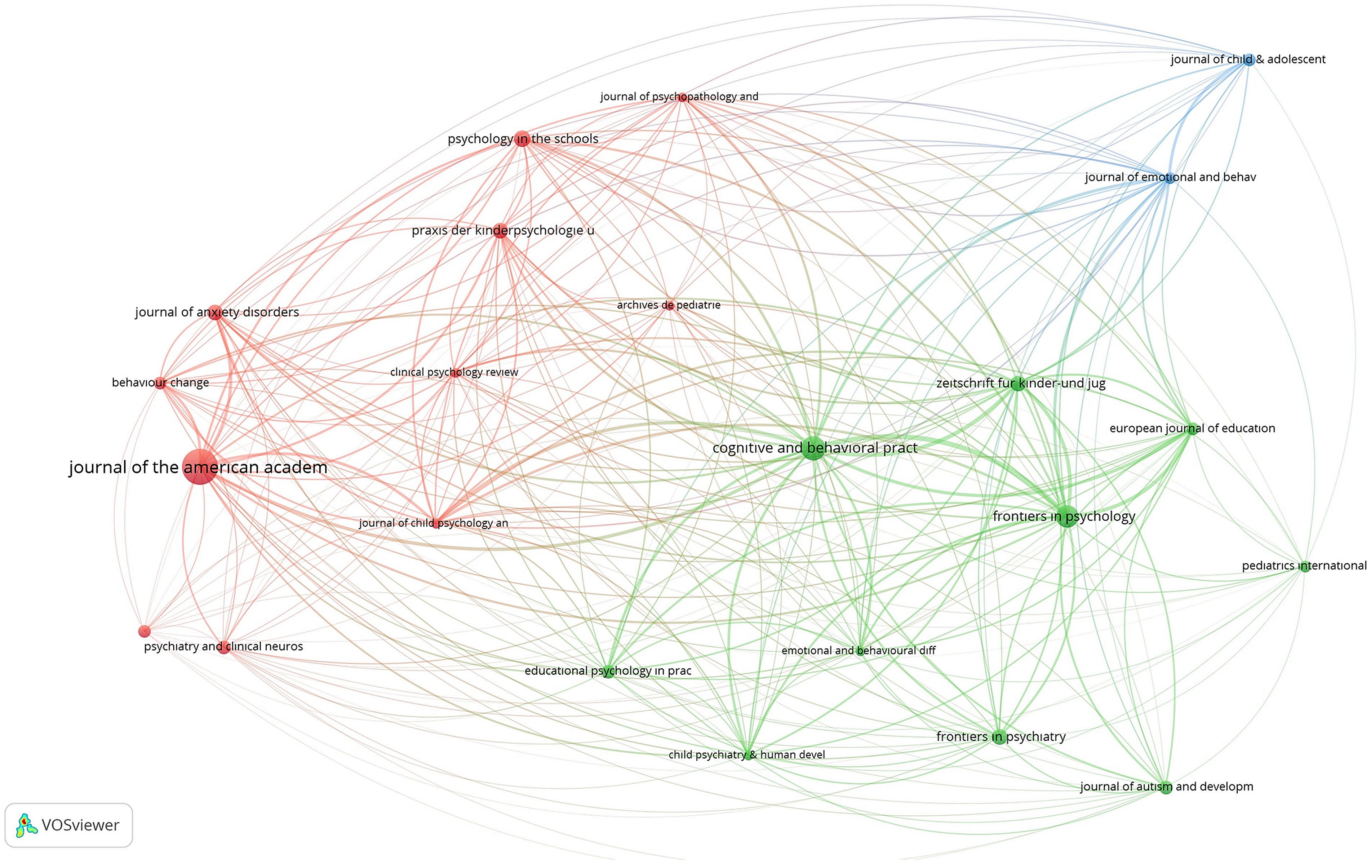
Visualization of the network of journals publishing SR studies.

**Figure 7:**
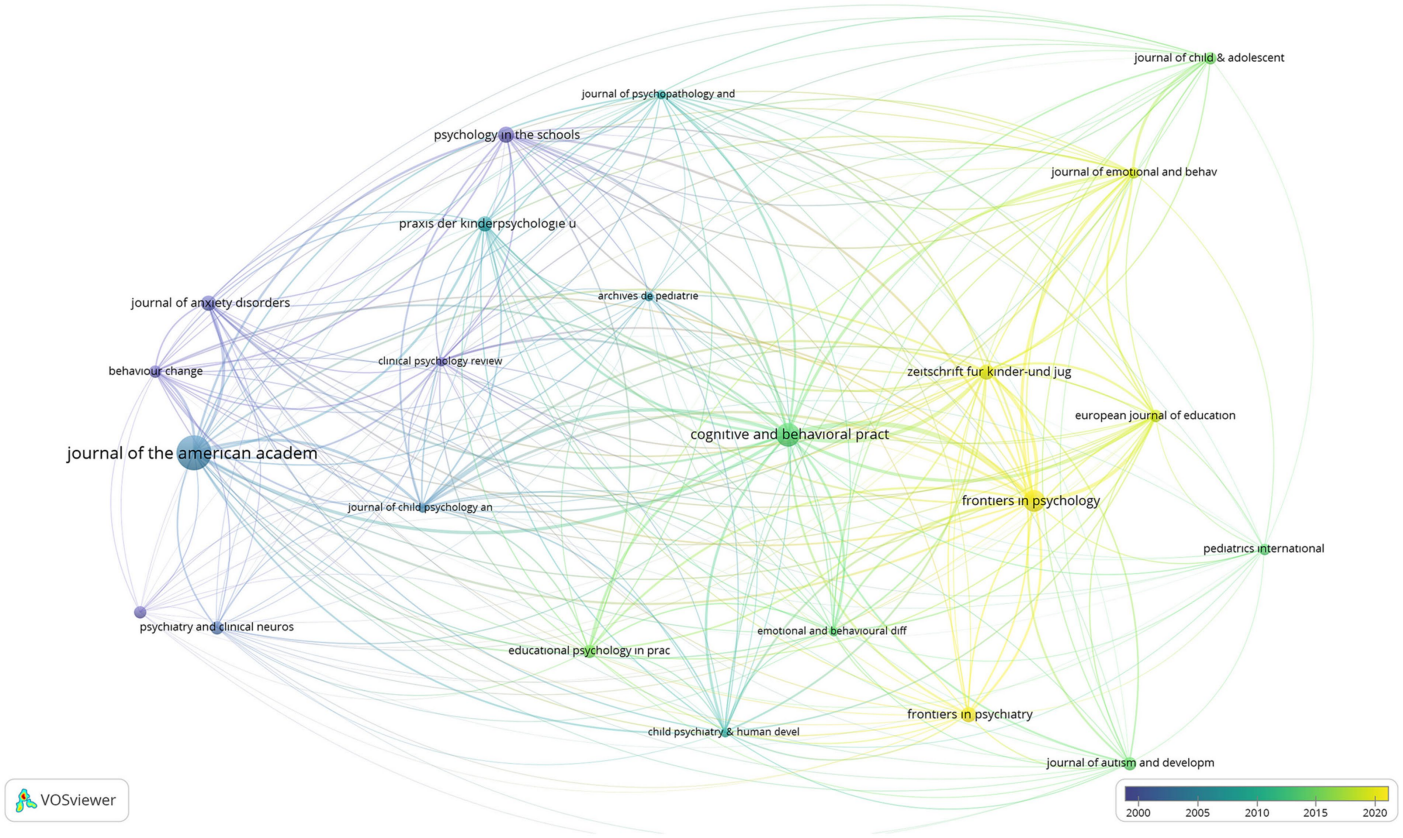
Visualization of the overlay of journals publishing SR studies.

In the analysis conducted to determine the journals in which studies on school refusal were published,

- The type of analysis: bibliographic coupling,
- The unit of analysis: sources,
- The min. Number of documents of a source:5,
- The min. Number of citations of a source:10.

were limited. Accordingly, 23 of the 310 sources identified as related are mapped as follows. In the mapping process, the number of documents was taken as the scale weight criterion and presented in Figures 6, 7.

Examining Figures 6, 7, it can be stated that the journals publishing SR studies are mapped in 3 clusters and 21 items. In this context, the items in the first cluster include Archives de Pediatrie (doc:5), Behavior Change (doc:7), British Medical Journal (doc:7), Clinical Psychology Review (doc:5), Journal of Anxiety Disorders (doc:9), Journal of American Academy of Child and Adolescent Psychiatry (doc:30), Praxis der Kinder-Psychologie und Kinder-Psychiatrie (doc:9), Psychiatry and Clinical Neuroscience (doc:8), Journal of Child Psychology and Psychiatry (doc:5), Journal of Psychopathology and Behavioral Assessment (doc:5), Psychology in the Schools (doc:10). Items in the second cluster are Child Psychiatry and Human Development (doc:5), Cognitive and Behavioral Practice (doc:17), Educational Psychology in Practice (doc:8), Emotional and Behavioral Difficulties (doc:5), European Journal of Education and Psychology (doc:7), Frontiers in Psychiatry (doc:9), Frontiers in Psychology (doc:15), Journal of Autism and Developmental Disorders (doc:8), Pediatric International (doc: 6), Zeitschrift fur Kinder und Jugend Psychiatrie und Psychotherapie (doc:9). Finally, Journal of Child and Adolescent Subtance Abuse (doc:7), Journal of Emotional and Behavioral Disorder (doc: 6) are the journal in the third cluster.

According to Figure 6, it can be said that the Journal of American Academy of Child and Adolescent Psychiatry, Cognitive and Behavioral Practice, and Frontiers in Psychology are important journals that publish on SR. According to Figure 7, it can be evaluated that Frontiers in Psychology, Frontiers in Psychiatry, Journal of Emotional and Behavioral Disorders, Zeitschrift fur Kinder und Jugend Psychiatrie und Psychotherapie, and European Journal of Education and Psychology are journals that make up-to-date publications on SR.

### 3.5 Scientific cooperation between researchers

**Figure 8:**
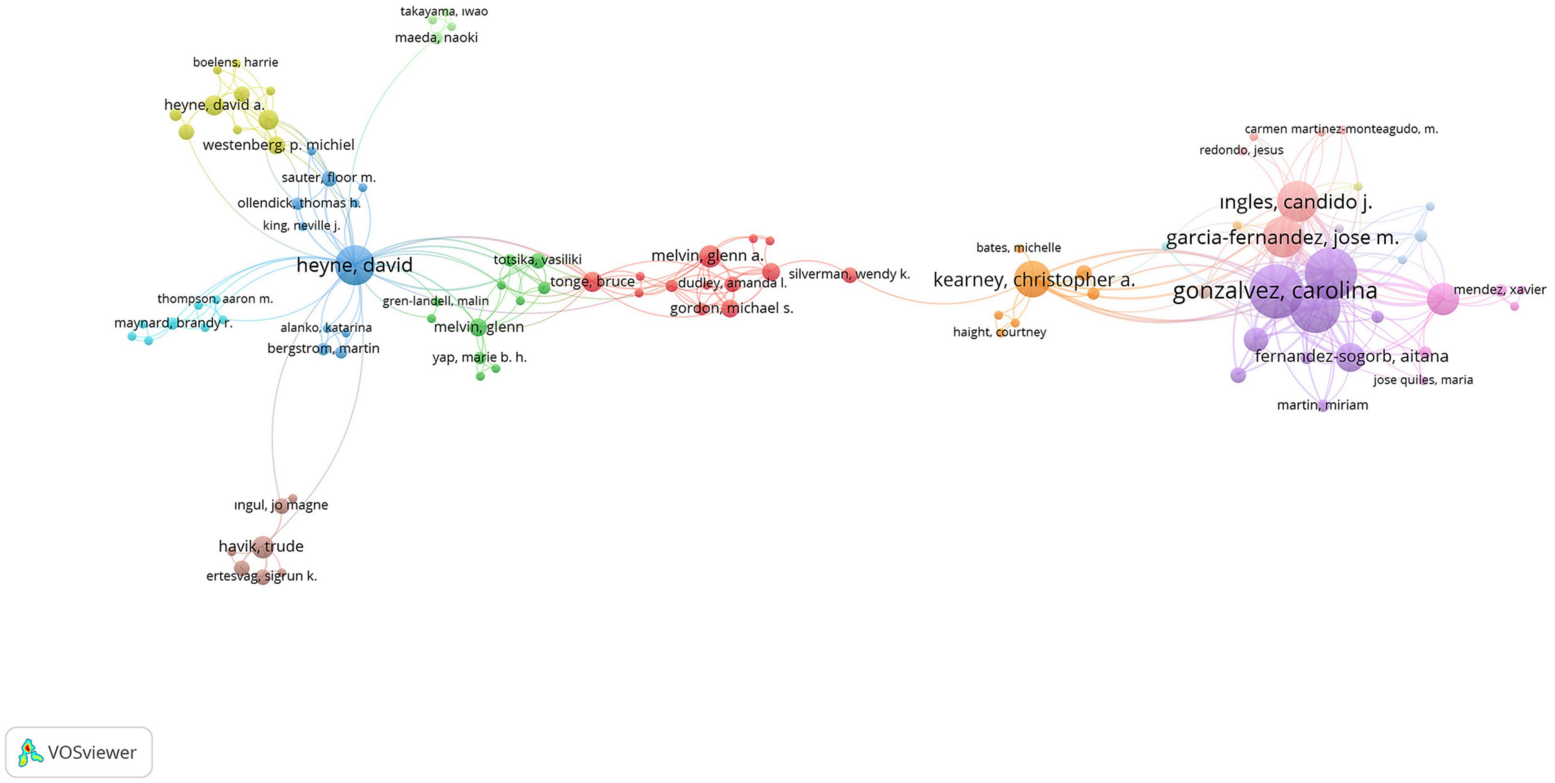
Visualization of SR researchers’ collaboration via networks.

**Figure 9:**
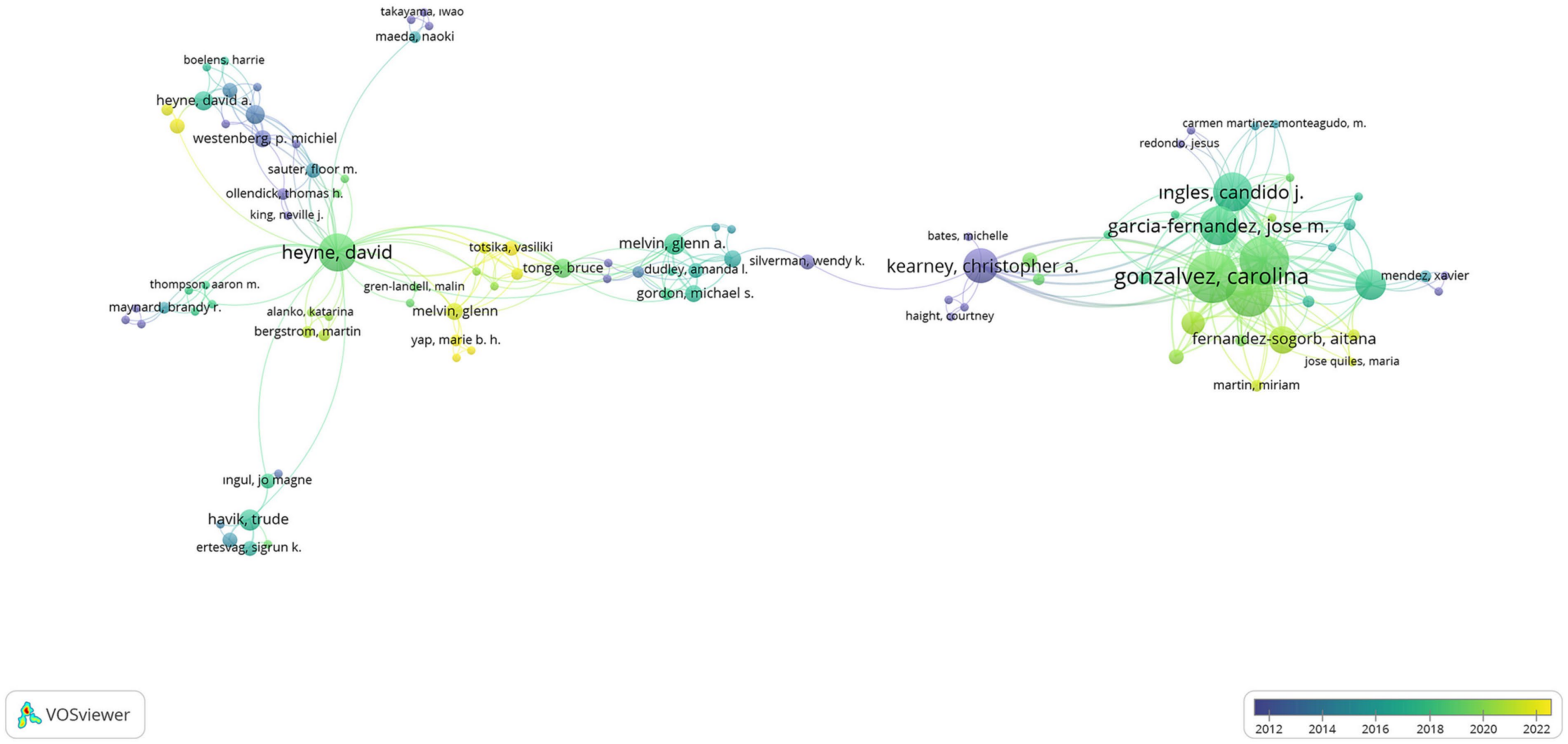
Visualization of SR researchers’ collaboration via overlay.

In order to determine the relationship patterns of the researchers included in this study on SR,

- The type of analysis: co-authorship,
- The unit of analysis: authors,
- The counting method: full counting,
- The min. Number of documents of an author: 1,
- The min. Number of citations of an Author: 5.

were limited. A total of 1,548 authors were analyzed depending on the limitations made by 1,150 authors, and the networks of 100 researchers were shown as visualization weight documents in Figures 8, 9, with the command to show the networks of relations with each other during visualization.

When Figure 8, which shows the relationships between researchers, is examined, it can be said that researchers named Christopher A. Kearney, Carolina Gonzalvez, Jose Manuel Garcia-Fernandez, David A. Heyne, Brigit M. Van Widenfelt, Angela Diaz-Herrero, Aitana Fernandez-Sogorb, Ricardo Sanmartin, Maria Vicent, Candido J. Ingles, and Bruce J. Tonge are prominent researchers in school refusal studies. According to Figure 9, it can be said that researchers named Martin Bergstrom, Vicki Bitsika, Katarina Alanko, Yoko Dutton, Vasiliki Totsika, Glenn Melvin, Richard P. Hastings, Aitana Fernandez-Sogorb, Miriam Martin, Maria Jose Quiles, Kylie Gray, Marie B. H. Yap, Meena Chockalingam, Kayan Skinner, Antonio M. Perez-Sanchez, and Mariola Gimenez-Miralles are open to scientific collaborations. In addition, the number of links, total link strengths, and documents reached as a result of the analysis of scientific collaborations between researchers is presented in Table 2 for all researchers.

**Table 2:** Number of links, total link strength, and document of researchers.

Cluster	Researcher	Link	Total link strength	Document
1. Cluster	Belinda Carless	3	3	1
	Amanda I. Dudley	10	16	4
	Michael S. Gordon	7	14	4
	Elenora Gullone	9	9	2
	Elizabeth K. Hughes	3	3	1
	Ester I. Klimkeit	6	6	1
	Gleen A. Melvin	12	21	6
	Louise K. Newman	3	3	1
	John Taffe	7	10	2
	Wendy K. Silverman	2	2	3
	Bruce J Tonge	23	34	9
2. Cluster	Meena Chockalingam	3	3	1
	Yoko Dutton	7	7	1
	Carolyn Gentle-Genitty	3	3	1
	Kylie Gray	7	10	2
	Malin Gren-Landell	3	3	1
	Richard P. Hastings	8	11	2
	Gleen Melvin	12	14	4
	Kayan Skinner	3	3	1
	Vasiliki Totsika	8	14	3
	Alison Worsley	7	7	1
	Marie B. H. Yap	3	4	2
3. Cluster	Katarina Alanko	4	4	1
	Martin Bergstrom	4	5	2
	Marije I. Brouwer-Borghuis	3	3	1
	David A. Heyne	40	59	24
	Neville J. King	2	2	1
	Thomas H. Ollendick	5	6	2
	Floor M. Sauter	7	11	3
	Ron H. J. Scholte	3	3	1
	Johan Strombeck	4	5	2
	Robin Ulriksen	4	4	1
	Robert Vermeiren	4	4	1
4. Cluster	Vicki Bitsika	3	5	3
	Harrie Boelens	4	4	1
	Peter De Heus	4	4	1
	David P. Mackinnon	4	4	1
	Marija Maric	7	12	3
	Christopher F. Sharpley	2	4	2
	Brigit M. Van Widenfelt	11	20	5
	Leonie J. Vreeke	4	4	1
	P. Michiel Westernberg	9	16	4
5. Cluster	Angela Diaz-Herrero	9	34	7
	Aitana Fernandez-Sogorb	10	39	10
	Carolina Gonzalvez	28	153	35
	Miriam Martin	5	8	2	
	Antonio Miguel Perez-Sanchez	11	23	5
	Cecilia Ruiz-Esteban	7	10	2
	Ricardo Sanmartin	21	136	29
	Maria Vicent	24	148	32
	Jose Manuel Garcia-Fernandez	35	142	32
6. Cluster	Kristen Esposito Brendel	5	5	1
	Jeffery J. Bulanda	5	5	1
	Brandy R. Maynard	8	8	2
	Kristen E. Peters	3	3	1
	Terri D. Pigott	5	5	1
	Christopher P. Salas-Wright	3	3	1
	Aaron M. Thompson	5	5	1
	Michael G. Vaughn	3	3	1
7. Cluster	Michelle Bates	1	1	1
	Mirae J. Fornander	3	6	2
	Patricia A. Graczyk	3	7	3
	Courtney Haight	3	3	1
	Marisa Hendron	3	3	1
	Christopher A. Kearney	14	34	16
	Rachel Schafer	3	3	1
8. Cluster	Edvin Bru	3	6	3
	Sigrun Ertesvag	5	8	4
	Trude Havik	6	11	6
	Jo Magne Ingul	3	4	3
	Maren Stahl Lerang	2	2	1
	Hans M. Nordahl	1	1	1
9. Cluster	Jose Pedro Espada	3	3	1
	Mariola Gimenez-Miralles	5	9	2
	Maria Jose Quiles	5	5	1
	Xavier Mendez	4	4	2
	Mireia Orgiles	3	3	1
10. Cluster	Maria Carmen Martinez-Monteagudo	7	7	2
	Candido J. Ingles	21	94	20
	Jesus Redondo	3	3	1
11. Cluster	Soichiro Hatada	3	3	1
	Naoki Maeda	4	4	2
	Junichi Sonoda	3	3	1
	Iwao Takayama	3	3	1
12. Cluster	David Aparisi-Sierna	6	6	1
	Beatriz Delgodo	5	5	1
	Maria Isabel Gomez-Nunez	7	11	2
13. And later cluster	Ainhoa Martinez-Palau	5	5	1
	Carlos M. Calderon	5	5	1
	Carlos E. Jimenez-Ayala	6	6	1
	Maria Del Pilar Aparicio-Flores	5	5	1
	Nelly Lagos-San Martin	7	12	2

### 3.6 Keywords of SR research

**Figure 10:**
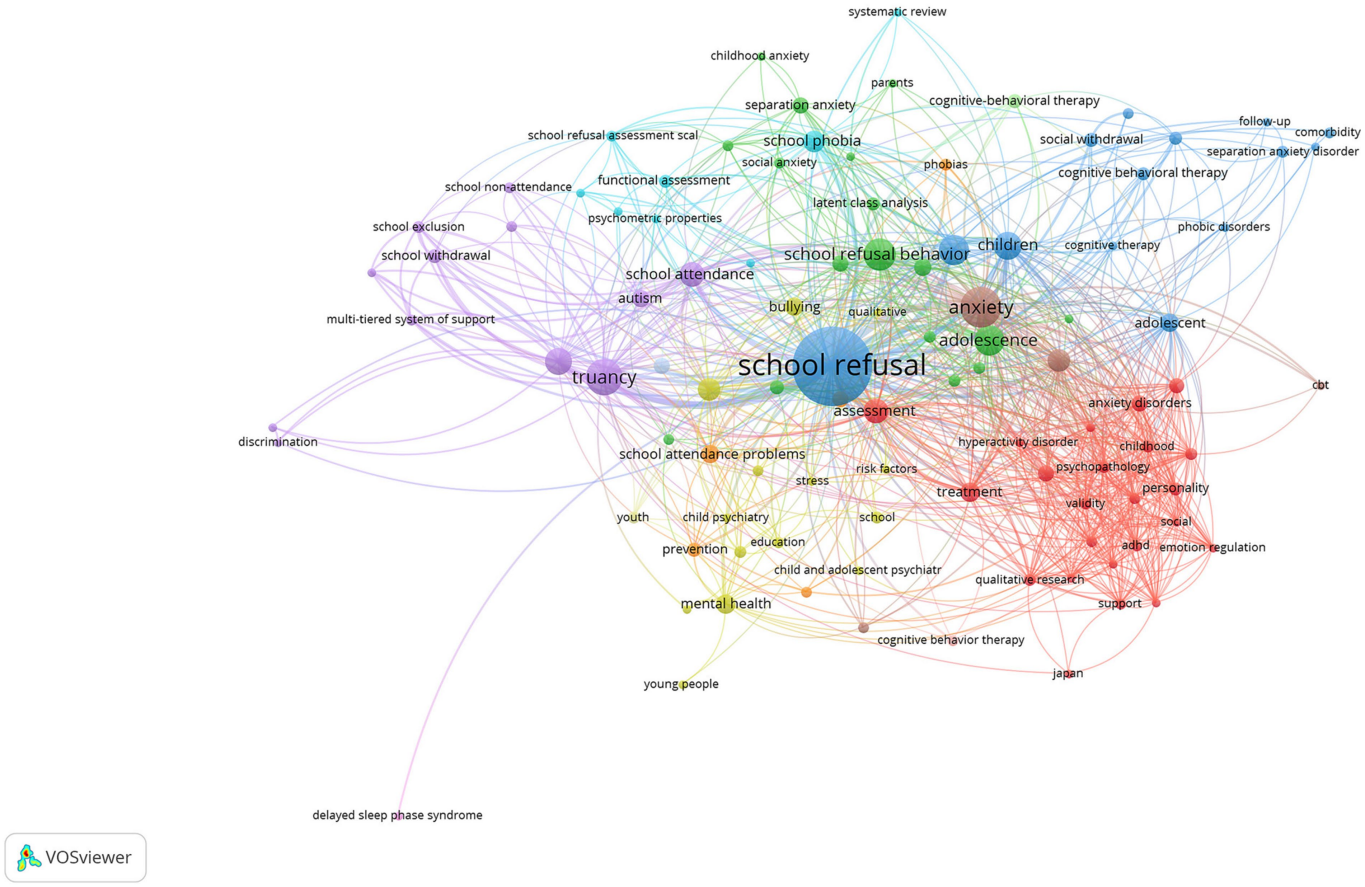
Visualizing the co-occurrence network of SR research keywords.

**Figure 11:**
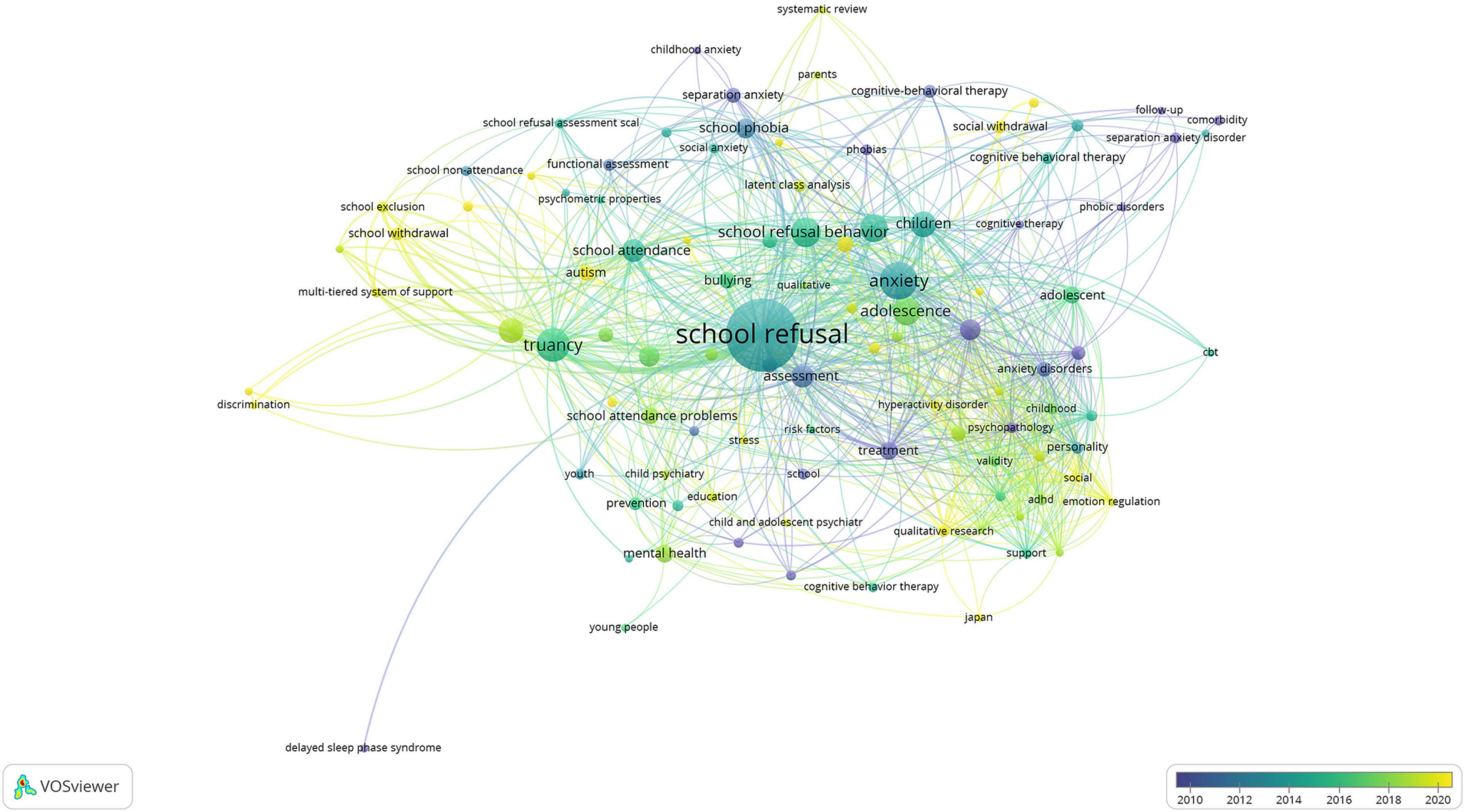
Visualizing the co-occurrence overlay of SR research keywords.

In order to create a co-occurrence map according to the author keywords of the studies on SR,

- The type of analysis: co-occurrence,
- The unit of analysis: author keywords,
- Counting method: full counting,
- The min. Number of occurrence of keywords: 3.

was determined. A total of 1,224 keywords were analyzed depending on the limitations and the networks of 102 keywords were shown in Figures 10, 11. Additionally, repetitive keywords (for example, adolescent, adolescents, adolescence) were used in a single cluster.

When the co-occurrence images of the keywords used in the studies on SR are examined (Figures 10, 11), the keywords in the 1st cluster; academic achievement (occurrences: 3), adhd (occurrences: 4), anxiety disorder (occurrences: 15), assessment (occurrences: 22), autism spectrum disorder (occurrences: 20), child (occurrences: 35), childhood (occurrences: 5), emotion regulation (occurrences: 3), hyperactivity disorder (occurrences: 3), Japan (occurrences: 3), parenting (occurrences: 3), personality (occurrences: 6), psychopathology (occurrences: 5), qualitative research (occurrences: 5), quality of life (occurrences: 4), review (occurrences: 3), self-efficacy (occurrences: 5), self-esteem (occurrences: 4), social (occurrences: 3), Spain (occurrences: 3), support (occurrences: 4), treatment (occurrences: 13), validity (occurrences: 5) was found. (2) Cluster academic self-attributions (occurrences: 3), adolescence (occurrences: 33), aggression (occurrences: 3), childhood anxiety (occurrences: 3), cluster analysis (occurrences: 7), factorial invariance (occurrences: 5), latent class analysis (occurrences: 6), latent profile analysis (occurrences: 11), parents (occurrences: 3), primary education (occurrences: 5), school absence (occurrences: 4), school anxiety (occurrences: 9), school refusal behavior (occurrences: 41), separation anxiety (occurrences: 15), social anxiety (occurrences: 7), and social functioning (occurrences: 5) keywords are included. (3) Cluster adolescents (occurrences: 43), cognitive behavioral therapy (occurrences: 9), cognitive therapy (occurrences: 3), comorbidity (occurrences: 4), follow-up (occurrences: 3), hikikomori (occurrences: 4), phobic disorder (occurrences: 3), school refusal (occurrences: 219), and social withdrawal (occurrences: 14) keywords has taken place. 4. cluster school absenteeism (occurrences: 18), bullying (occurrences: 12), child and adolescent psychiatry (occurrences: 3), child psychiatry (occurrences: 3), delinquency (occurrences: 3), education (occurrences: 4), mental health (occurrences: 13), qualitative (occurrences: 3), risk factors (occurrences: 3), school (occurrences: 5), school health (occurrences: 4), stress (occurrences: 3), substance use (occurrences: 5), and young people (occurrences: 3) were included. (5) Cluster includes discrimination (occurrences: 3), intellectual disability (occurrences: 4), minorities (occurrences: 3), multi-tiered system of supports (4), response to intervention (occurrences: 3), school absenteeism (occurrences: 26), school attendance (occurrences: 22), school exclusion (occurrences: 5), school non-attendance (occurrences: 4), and truancy (occurrences: 48) keywords. (6) Cluster; attention deficit (occurrences: 3), functional assessment (occurrences: 6), psychometric properties (occurrences: 3), school attendance problem (occurrences: 15), school phobia (occurrences: 16), school refusal assessment scale/revised form (occurrences: 7) keywords are included. (7) The cluster contains keywords epidemiology (occurrences: 4), intervention (occurrences: 10), phobias (occurrences: 5), and prevention (occurrences: 7). Cluster 8 includes anxiety (occurrences: 59), depression (occurrences: 19), and family (occurrences: 4), while youth (occurrences: 5), delayed sleep phase syndrome (occurrences: 3), and school avoidance (occurrences: 9) are included in Cluster 9 and later cluster.

Examining Figure 10, one of the author keywords on SR, truancy, school absenteeism, adolescence, school phobia, autism spectrum disorder, treatment, separation anxiety, depression, intervention, mental health, latent profile analysis, school attendance problem, assessment, functional assessment, anxiety, psychopathology, school avoidance, social withdrawal, social functioning, and cognitive behavior therapy keywords become prominent. Examining Figure 11, it can be said that the keywords of school withdrawal, stress, school exclusion, multi-tiered system of support, discrimination, autism spectrum disorder, social withdrawal, stress, parents, functional assessment, academic self-attributions, self-esteem, attention deficit, intellectual disability, hyperactivity disorder, emotional regulation, social functioning, academic achievement, systematic review, and qualitative research are currently keywords compared to others.

## 4 Discussion

The present study aims to determine research trends and scientific collaborations on school refusal by using bibliometric data obtained from the WoS database. Depending on this purpose, the results obtained from the analysis on publication year of SR publications, countries, organizations, researchers, journals of publications, and key concepts are presented in the result section. As a result of the analysis, school refusal publications tend to increase over the years, and the United States was positioned as a central node in the studies on school refusal (Kearney and Silverman, 1990; Kearney, 1993; Fremont, 2003; Kearney and Albano, 2004; Kearney, 2006; Hilt, 2014; Ye et al., 2017; Londono Tobon et al., 2018; Li et al., 2021; McClemont et al., 2021). Historically, Japan (Fukuda and Hozumi, 1987; Hoshino et al., 1987), Australia (King and Ollendick, 1989; Pritchard et al., 1998; Tonge, 1998), Canada (Rubenstein and Hastings, 1980; Atkinson et al., 1989), New Zealand (Stein et al., 2001), England (Berg, 1985; Foreman et al., 1997; Wray and Thomas, 2013; Katz et al., 2016), it seems that there are studies on school refusal in their countries. The review study conducted by Kearney (2008) stated that similar studies on school attendance problems and school refusal were undertaken primarily in the United States, the United Kingdom, Canada, and Australia. However, when the current studies on school refusal are examined, Spain (Inglés et al., 2015; García-Fernández et al., 2016; Gonzálvez et al., 2016; Sanmartín et al., 2018; Gonzálvez et al., 2020), Norway (Havik et al., 2014; Munkhaugen et al., 2019; Havik and Ingul, 2021; Høiseth et al., 2021), Finland (Strömbeck et al., 2021), Belgium, (Al-Husni Al-Keilani and Delvenne, 2021), and Turkey (Bahali et al., 2009; Oner et al., 2014; Secer, 2014; Seçer and Ulaş, 2020) countries seem to be gaining momentum. As expected, this situation is similar in terms of the universities where school refusal studies are carried out, and University of Alicante, Central University of Ecuador, University Illinois, University Bristol, Leiden University, and University College London, Monash University, and University Paris universities are at the forefront of current studies.

As a result of the analysis of the keywords used in studies on school refusal, it is clear that the keywords that came to the fore in the general pattern include school refusal, truancy, school absenteeism, adolescence, school attendance, school phobia, autism spectrum disorder, bullying, treatment, school anxiety, separation anxiety, depression, intervention, mental health, latent profile analysis, school attendance problem, assessment, functional assessment, anxiety, psychopathology, self-efficacy, school avoidance, social withdrawal, social functioning, and cognitive behavior therapy; however, in the current pattern, it was observed that the keywords of school withdrawal, stress, school exclusion, autism spectrum disorder, hyperactivity disorder, emotional regulation, social functioning, academic achievement, systematic review, and qualitative research came to the forefront. Among the oldest topics studied chronologically in WoS, school refusal includes depression, childhood anxiety, and phobic disorders. In this context, Ingul et al. (2019) emphasized in a previous study that depression, anxiety, and somatic symptoms are important warning signals for school refusal. Similarly, Gonzálvez et al. (2018) discussed the relationship between school refusal and depression, anxiety, and stress. The results revealed a relationship between the variables that pointed to negative affectation and school refusal. At this point, the keywords of emotion regulation, and the current concept of school refusal in WoS, drew attention. Indeed, emotion regulation difficulties are considered an individual risk factor for school refusal (Filippello et al., 2018). In line with all these evaluations, Heyne (2022) suggested that emotion regulation skills should be included in the intervention of school refusal. In a study conducted by Carpentieri et al. (2022), adolescents seeking psychological help were grouped as those with school refusal and those without school refusal. In the grouping, anxiety, and depression levels were higher than those in the group without school refusal, and emotion regulation and social functioning levels were reported to be lower. At this point, it is seen that one of the keywords that come to the forefront of school refusal research is social functioning, which has been evaluated to have a protective function against school refusal (Gonzálvez et al., 2019; Seçer and Ulaş, 2020). In a study of children with autism, the sample structure was similar to that of the group with and without school refusal, and it was found that they were at a lower level in terms of social behaviors (initiating any activity, reacting, etc.) and had a more depressive structure (Munkhaugen et al., 2019). In another study, children with typical development and school refusal were examined comparatively, and children with an autism diagnosis and school refusal behavior were examined comparatively. It was stated that school refusal behaviors appeared earlier in children with autism. Accordingly, it is recommended that evaluations of children with autism should be made at an early stage (Ochi et al., 2020).

Children with attention deficit and hyperactive disorder in the high-risk group for school refusal in the literature (McClemont et al., 2021; Granieri et al., 2022) were found to be the current keyword in this study. It was reported in a study carried out by Dan and Benovich (2022) that male adolescents with ADHD had low school attendance rates and intense school refusal behaviors. It was stated that their negative attitudes towards school and anxiety levels were higher than their peers with typical development. It was found that adolescents with ADHD should progress based on anxiety in the process of working with school refusal behaviors.

## 5 Conclusion

As a result of the bibliometric analysis of the data obtained from the WoS database, it was seen that the issue of SR emerged in the 1970s and had a stable publication potential until 1995, and in 2012–2023, 57% of the studies carried out so far were done. Accordingly, it can be said that the issue of SR attracts the attention of researchers as a current research area. The studies carried out were largely in the United States, Japan, Spain, England, Norway, Finland, Belgium, Turkey, Malaysia, Spain, and University Nevada, Virginia Polytech Institute, State University, Monash University, University Minnesota, Florida International University, University of Alicante, Leiden University, University of Pennsylvania, Miguel Hernandez University of Elce Spain, Temple University, New York University, University Hawaii Manoa, University Utrecht, University Stavanger, University of California Los Angeles, John Hopkins University, University of Alicante, Central University of Ecuador, University Illinois, University Bristol, Leiden University, University College London, Monash University, and University Paris. The journals in which the most published denial research was found were the Journal of American Academy of Child and Adolescent Psychiatry, Cognitive and Behavioral Practice, Frontiers in Psychiatry, Frontiers in Psychology, Zeitschrift fur Kinder und Jugend Psychiatrie und Psychotherapie, and the European Journal of Education and Psychology. Prominent researchers in the field of school refusal were Christopher A. Kearney, Carolina Gonzalvez, Jose Manuel Garcia-Fernandez, David A. Heyne, Bruce J. Tonge, Martin Bergstrom, Robert Palmer, Vicki Bitsika, Katarina Alanko, Yoko Dutton, and Mariola Gimenez-Miralles. As a result of the analysis was conducted to determine the keywords of school refusal, truancy, school absenteeism, adolescence, school attendance, school phobia, autism spectrum disorder, bullying, treatment, school anxiety, separation anxiety, depression, intervention, mental health, latent profile analysis, school attendance problem, assessment, functional assessment, anxiety, psychopathology, self-efficacy, school avoidance, social withdrawal, social functioning, and cognitive behavior therapy, school withdrawal, stress, school exclusion, autism spectrum disorder, hyperactivity disorder, emotional regulation, social functioning, academic achievement, systematic review, and qualitative research.

## 6 Limitation

A limitation in this study is that the keywords were searched only in the topic. Obtaining the data only from WoS database is another limitation because it might not offer access to some of the scientific literature. The results of bibliometric analyses may differ in terms of specific factors such as search engine, keywords, differences in listing the author names on the article, and the use of “or” or “and” together. Moreover, the search location of the keywords in the study (e.g., the topic was used in the present study), year of publication, language, etc. will also affect the bibliometric data available. Since VOSviewer program was used in the analyses, the method used for examining the repetition of items can be considered another limitation. There might be errors in the names of universities and researchers, as well as keywords. On the other hand, the strengths of this bibliometric analysis are the visualization of data such as prominent keywords, countries, universities, researchers, and journals which exhibit general patterns associated with the historical changes in SR.

The results achieved in this study are very important in terms of possible scientific collaborations, especially for researchers, by providing a picture of the research centers/universities and researchers that come to the fore regarding SR. For educators and experts in the field of psychology, examining the variables related to school refusal and variables that can be grouped such as risk factors and reasons for school refusal are important for the process of preparing intervention and psychoeducational content for school refusal.

## Data availability statement

The raw data supporting the conclusions of this article will be made available by the authors, without undue reservation.

## Author contributions

SU: Conceptualization, Data curation, Formal analysis, Investigation, Methodology, Software, Validation, Visualization, Writing – original draft, Writing – review & editing. CG: Conceptualization, Funding acquisition, Investigation, Project administration, Supervision, Writing – original draft, Writing – review & editing. İS: Conceptualization, Investigation, Project administration, Resources, Supervision, Writing – original draft, Writing – review & editing.
